# The Use of Proton Pump Inhibitors and COVID-19: A Systematic Review and Meta-Analysis

**DOI:** 10.3390/tropicalmed7030037

**Published:** 2022-02-28

**Authors:** Kaneez Fatima, Talal Almas, Shan Lakhani, Arshia Jahangir, Abdullah Ahmed, Ayra Siddiqui, Aiman Rahim, Saleha Ahmed Qureshi, Zukhruf Arshad, Shilpa Golani, Adeena Musheer

**Affiliations:** 1 Department of Medicine, Dow University of Health Sciences, Karachi 75510, Pakistan; kaneezfatima344@gmail.com (K.F.); aimanrahim2001@gmail.com (A.R.); zukhrufarshad200@gmail.com (Z.A.); adeenamusheer06@gmail.com (A.M.); 2 Department of Medicine, Royal College of Surgeons in Ireland, University of Medicine and Health Sciences, 15503 Dublin, Ireland; 3 Department of Medicine, The Aga Khan University, Stadium Road, Karachi 74800, Pakistan; shan.lakhani@scholar.aku.edu (S.L.); arshia.jahangir@scholar.aku.edu (A.J.); abdullah.ahmed@scholar.aku.edu (A.A.); ayra.siddiqui@scholar.aku.edu (A.S.); saleha.qureshi@scholar.aku.edu (S.A.Q.); shilpa.golani@scholar.aku.edu (S.G.)

**Keywords:** corona virus, COVID, proton pump inhibitor, severity, susceptibility, infection

## Abstract

COVID-19 has proved to be a serious, and consequential disease that has affected millions of people globally. Previously, the adverse effects of proton pump inhibitors (PPI) have been observed with increasing the risk of pneumonia and COVID-19. This meta-analysis aims to address the relationship between the use of PPI and the severity of COVID-19 infection. We conducted a systemic literature search from PUBMED, Science Direct, and Cinahl from December 2019 to January 2022. Published and unpublished randomized control trials and cohort studies were included. Review Manager was used for all statistical analyses. In total, 14 studies were included in this systemic review and meta-analysis. Outcomes of interest include: (1) susceptibility of COVID-19 infection and (2) severity of COVID-19 (defined as the composite of poor outcomes: ICU admission, need for oxygen therapy, need for a ventilator, or death), and (3) mortality due to COVID-19. PPI use was marginally associated with a nominal but statistically significant increase in the risk of COVID-19 infection (OR 1.05 [1.01, 1.09]; I^2^ 97%, *p* = 0.007). PPI use also increased the risk of the composite poor outcome (OR 1.84 [1.71, 1.99]; I^2^ 98%, *p* < 0.00001) and mortality (OR 1.12 [1.00, 1.25]; I^2^ 84%, *p* = 0.05) in patients with COVID-19.

## 1. Introduction

Coronavirus disease 2019 (COVID-19) is a highly contagious viral illness that has been responsible for the loss of 5.3 million lives globally (as of December 2021) [1]. To control this pandemic, it is crucial to seek preventative methods by identifying any risk factors for increased morbidity or mortality in COVID-19 infection. It has been shown that routinely used medications can variably influence the susceptibility to COVID-19 infection, as well as the severity of the disease. Treatment of COVID-19 infection with antihistamines and azithromycin, in symptomatic as well as asymptomatic and susceptible patients, is associated with reduced severity and mortality—irrespective of patients’ age and risk factors [2]. ACE inhibitors and ARBs appear to have no effect on the incidence and prognosis of COVID-19 [3]. Systemic corticosteroids and biologics used in treating asthmatic patients are not associated with increased susceptibility to COVID-19 infection [4]. On the other hand, the adverse effects of opioids can potentially increase the severity of COVID-19 infection, especially by lowering the immune response [5]. Obesity is also another risk factor for worsening the COVID-19 disease across various ethnicities [6]. In addition, hypertensive patients had been observed to have a higher proportion of pulmonary infection and an increased risk for severe adverse COVID-19 outcomes [7].

Proton pump inhibitors (PPI) are chiefly prescribed over the counter for upper gastrointestinal acid-related disorders, such as gastroesophageal reflux disease (GERD), non-erosive reflux disease, and peptic ulcer disease. Due to the high prevalence of upper GI acid-related disorders, high efficacy, favorable safety profile, and affordability, PPI are prescribed frequently and at higher doses for longer durations [8]. In fact, PPI are one of the top ten most used drugs in the world and are prescribed without any clear indication in up to 70% of cases [9]. PPI can negatively affect several organ systems in the body. For instance, associations have been found between PPI and an increased risk of developing chronic kidney disease, which can progress to end-stage renal disease and pneumonia due to PPI-induced hypochloremia [10,11]. Due to the inhibition of acid secretion, hepatotoxicity, neuroendocrine neoplasms, and cancers in the gastrointestinal tract have also been reported with PPI administration [12,13,14].

Given that several medications have been shown to influence COVID-19 incidence and outcomes, and that PPI have systemic effects—it is essential to investigate the relationship between PPI use and COVID-19 susceptibility and outcomes. Previous studies have shown inconsistent associations [15,16]. For instance, Almario et al. [16] reported an increase in the likelihood of developing COVID-19 infection after PPI administration. In contrast, Xiang Y et al. [17] concluded that PPI decrease the susceptibility to COVID-19. Furthermore, Ramchandran et al. [18] observed increased mortality with PPI usage whereas Fan X [19] did not find an association between PPI and mortality due to COVID-19 infection. This meta-analysis was conducted in order to resolve these inconsistencies and provide a holistic, well-powered assessment of the effect of PPI use on the incidence and prognosis of COVID-19.

## 2. Materials and Methods

### 2.1. Data Sources and Search Strategy

We undertook a systematic review and meta-analysis investigating the effects of PPI in patients diagnosed with COVID-19. Articles eligible for inclusion were observational cohort, case control, or randomized controlled trials (RCTs) characterizing severity and mortality of COVID-19 infection in patients on PPI therapy. This meta-analysis was carried out as per the recommended guidelines for systematic review and meta-analyses in the Preferred Reporting Items for Systematic Reviews and Meta-Analyses (PRISMA) guidelines [20]. We designed a high sensitivity search strategy, Supplementary Table S1 [21], combining free text and keyword (COVID-19, Coronavirus, Proton pump inhibitor, severity, morbidity, mortality) search term synonym clusters for COVID-19, combined with clusters for PPI.

PUBMED, Cinahl, and Science Direct were screened from December 2019 to January 2022, using the various names of all PPI and keywords related to COVID-19. The retrieved articles were manually screened to identify relevant studies.

### 2.2. Study Selection

The following inclusion criteria were used to select studies: (a) published and unpublished cohort and case control studies linking PPI usage to outcomes of COVID-19 infection; (b) published or unpublished cohort or case control studies linking PPI use with COVID-19 severity or mortality. For inclusion, the studies had to report at least one of the following outcomes of interest: (1) susceptibility of COVID-19 infection, and (2) severity of COVID-19 (defined as the composite of poor outcomes: ICU admission, need for oxygen therapy, need for a ventilator, or death), and (3) mortality due to COVID-19.

The following exclusion criteria were used: (a) commentaries, perspectives, and editorials that give a subjective impact of PPI on the topic without clinical patient data were excluded; (b) articles that were not in the English language.

In total, 14 studies fulfilled the eligibility criteria and were included in this meta-analysis. The characteristics of the included studies are mentioned in Table 1, and the baseline characteristics of the patients and their PPI usage are mentioned in Table 2 and Table 3, respectively.

### 2.3. Data Extraction and Assessment of Study Quality

EndNote Reference Library was used to remove duplicates. Next, the articles were thoroughly screened and evaluated by two reviewers (AS and SL), and only those studies that fulfilled the aforementioned criteria were included. Firstly, studies were shortlisted based on their titles and abstracts, followed by a detailed review of the full article to ensure relevance. Then, a third investigator (AJ) was consulted to resolve any discrepancies. The studies yielded by our search strategy did not include any randomized control trials (RCTs). The Newcastle-Ottawa Scale was used to assess the quality and risk of bias for cohorts and case control studies, Supplementary Table S2 [21]. Studies were rated on a scale of 0–9. Studies with a cumulative score of 5 or less were considered to be low quality, those with a score of 6–7 were considered to be of moderate quality, and a study with a score of 8 or more was considered to be of high quality. Only moderate and high quality studies were included in our analysis. 

### 2.4. Statistical Analysis

A statistical analysis was performed using Review Manager (version 5.3). The outcomes from the studies were stated as odds ratios (ORs) with 95% confidence intervals (CIs) and were pooled using a random-effects model. Forest plots were generated to visually represent the results. The chi-square test was performed to assess for differences between the subgroups. Heterogeneity across studies was evaluated using Higgins I^2^ [31]. For I^2^, values from 25–50% indicated low heterogeneity, 50–75% indicated moderate heterogeneity, and more than 75% indicated severe heterogeneity. A *p*-value of ≤0.05 was regarded as significant in all cases.

## 3. Results

### 3.1. Search Result

**Figure 1:**
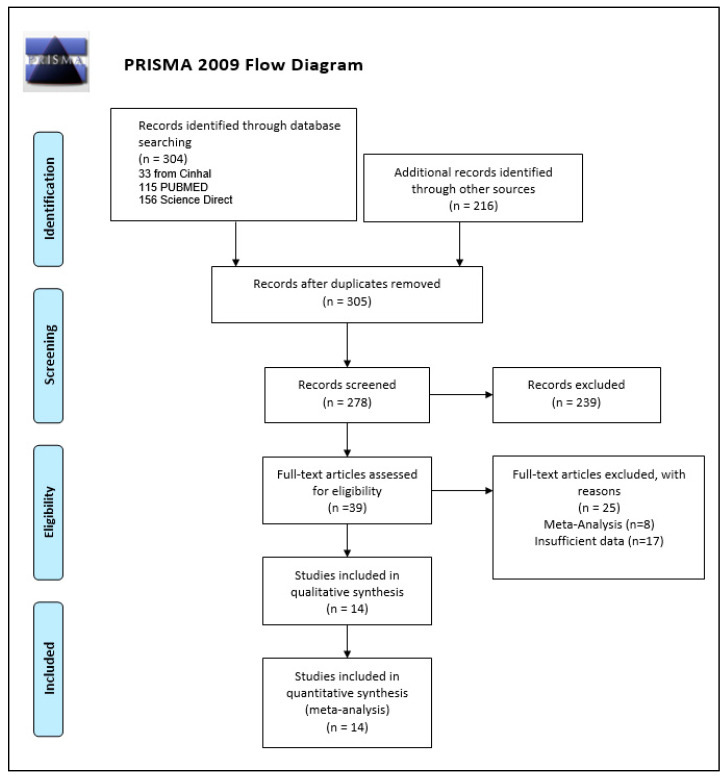
Prisma flow diagram.

Our search string produced 304 studies from our primary databases (33 from Cinahl, 115 PUBMED, 156 Science Direct), additional records of 216 studies were also identified through other sources. After an exclusion of duplicates, 278 studies were screened which yielded a total of 39 studies for full assessment. Of these, eight studies were excluded for being previous meta-analyses and another 17 were removed for not having sufficient data to permit calculations. The remaining studies were carefully evaluated in detail. The final number of studies included in the qualitative synthesis and quantitative synthesis were (n = 14). The PRISMA flow diagram of the selection process is provided in Figure 1.

### 3.2. Study Characteristics and Quality Assessment

Out of the 14 studies we selected for this meta-analysis and systematic review, 10 were cohort studies with 461,003 patients in total, and the other four of them were case control, which included one retrospective case control and one matched case control, with 16,154 patients in total. From the 14 articles, a total of 477,157 patients with laboratory-confirmed COVID-19 infection were identified. Individual study characteristics and patient demographics are presented in Table 1 and Table 2, respectively. The Newcastle-Ottawa Scale (NOS) (range from 0 to 9 points) was used to evaluate quality and risk of bias for all the 14, non-randomized included studies, Supplementary Table S2 [21]. The risk of bias appeared higher for some included studies in comparison to others; however, no important difference was noted in sensitivity analyses by excluding studies at higher risk of bias. Quality assessment was completed independently by two reviewers, and any disagreements were settled by discussion.

### 3.3. Outcomes

#### 3.3.1. PPI Usage and Susceptibility to COVID-19

**Figure 2:**
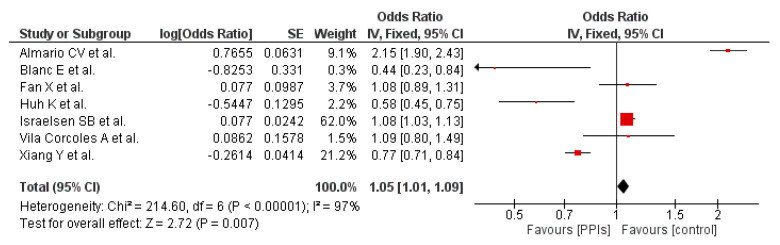
Probability of developing COVID-19 in patients on PPI versus not on PPI.

Seven out of the 14 included studies provided evidence for this outcome. Overall, our pooled analysis shows that PPI use results in a nominal but statistically significant increased risk of developing COVID-19 (OR 1.05 [1.01, 1.09]; I^2^ 97%, *p* = 0.007), Figure 2. In the forest plots, the individual point estimates for each study are illustrated by blocks and lines and the diamond represents the meta-analysis of each outcome using the chosen effect measure [32].

#### 3.3.2. PPI Usage and Composite Poor Outcome

**Figure 3:**
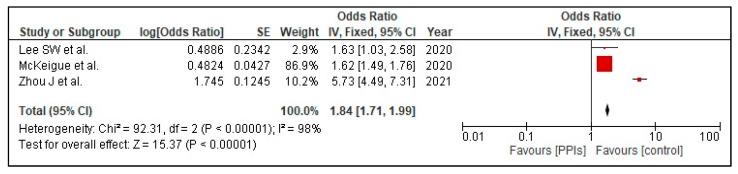
Risk of poor composite outcome in patients with COVID-19 on PPI versus not on PPI.

The composite poor outcome (ICU admission, need for oxygen therapy, need for a ventilator, or death) was reported in three studies, [22,23,24]. Our pooled analysis shows that PPI use is associated with a statistically significant increase in the risk of the composite poor outcome (OR 1.84 [1.71, 1.99]; I^2^ 98%, *p* < 0.00001), Figure 3.

#### 3.3.3. PPI Usage and Mortality

**Figure 4:**
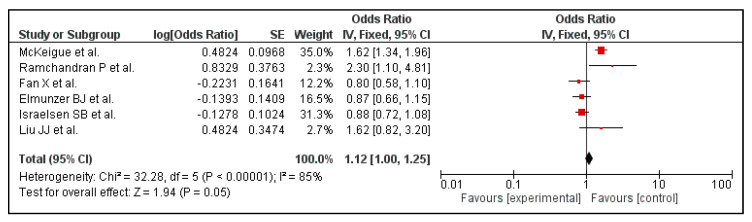
Risk of mortality in patients with COVID-19 on PPI versus not on PPI.

Six out of the 14 included studies reported the association between PPI use and mortality. Before carrying out a leave-one-out sensitivity analysis, the association between use of PPI and mortality was found to be borderline significant (OR 1.12 [1.00, 1.25]; I^2^ 85%, *p* = 0.05), Figure 4.

### 3.4. Publication Bias

As all the outcomes included studies less than 10, a funnel plot was not retrieved, and publication bias was not assessed [33].

### 3.5. Sensitivity Analysis

**Figure 5:**
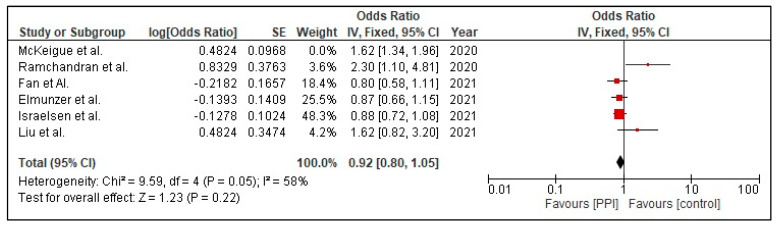
Risk mortality in patients with COVID-19 on PPI versus not on PPI after sensitivity analysis.

A sensitivity analysis was performed in order to tackle heterogeneity. This was performed for the outcome of mortality in COVID-19 patients with PPI use. One of the studies was excluded, namely McKeigue et al. [26]. Then, the analysis was repeated to obtain a forest plot (Figure 4). This sensitivity analysis brought a change in the final odds ratio from (OR 1.12 [1.00, 1.25] *p* = 0.05) to (OR 0.92 [0.80, 1.05] *p* = 0.22), Figure 5, for the outcome of mortality. The heterogeneity reduced from I^2^ = 85% to a moderate value of I^2^ = 58% when the study was excluded.

## 4. Discussion

This is the largest meta-analysis conducted on this topic comprising a total of 477,157 COVID-19 positive patients from 14 studies. According to our results, PPI use is associated with a nominal but statistically significant increase in the risk of developing COVID-19. Moreover, PPI use increased the severity of COVID-19 infection (i.e., the combined risk of ICU admission, need for oxygen therapy, mechanical ventilators, or death). Additionally, we found a borderline significant association between PPI use and mortality in patients with COVID-19.

A 2020 meta-analysis conducted by Israelson et al. [15] showed that PPI increased the risk of COVID-19 infection. An update of this analysis in 2021 by Pranta et al. [34] demonstrated no significant relationship between PPI use and susceptibility to COVID-19. The current meta-analysis consolidates the idea that PPI use is associated with an increased risk of developing COVID-19; however, the risk is only marginal. A recent meta-analysis by Hariyanto et al. [35] correlated with an increased risk of secondary outcomes such as ARDS, gastric tumors, cardiovascular diseases, and kidney diseases. Decreased enzymatic activity of dimethylarginine dimethylaminohydrolase (DDAH) inhibits nitric oxide synthase leading to thrombosis which is a potential cause of cardiovascular diseases. PPI have also been reported to cause nephritis and a humoral and cell-mediated hypersensitivity reaction. The aforementioned events contribute to the severe outcomes and mortality as a result of COVID-19 infection. Inhibition of neutrophil function causes a decrease in the eradication of the virus thus increasing the severity of infection. Irreversible proton pump inhibition is the primary effect of PPI, which diminishes gastric output in the body. Gastric acid is a partial barrier that limits the entry of SARS-CoV-2 viruses into the remaining gastrointestinal tract. When this restrictive acid barrier is withdrawn, by a PPI dosage, it raises the gastric pH to 6.0 from its normal levels of 1.5–3 [36]. The hypochlorhydria subsequent to PPI use is linked with a greater risk of susceptibility for viral infections like COVID-19. Prolonged survival of the COVID-19 virus in the stomach of individuals consuming PPI creates better provision for invasion of epithelial cells in the gastrointestinal tract. Excessive suppression of gastric acid due to PPI also means ingested pathogens are not readily removed from the stomach, with alteration of various immune-modulatory and anti-inflammatory effects [37]. It has been established that the COVID-19 virus, using the spike-like proteins on its surface, binds to ACE-2 of alveolar type 2, intestinal, and other types of cells [38]. This means that a higher expression of ACE-2 will lead to greater binding with the virus, and thus, a higher level of viral entry into the cell causing much more severity of the infection [39]. The GI tract demonstrates greater levels of ACE-2, hence individuals using PPI may be more vulnerable to infection with lower viral loads [38]. This idea is supported by the link between PPI use and increased risk of other respiratory infections—in a meta-analysis of clinical trials conducted by Nabil Sultan et al. the absolute incidence of respiratory infections was estimated to be 1.3% higher in patients receiving PPI than in those receiving a placebo [40].

This study also demonstrates an association between PPI use and adverse clinical outcomes in patients with COVID-19. PPI may increase the severity of COVID-19 by promoting suppression of the immune system [41]. Previously present experimental data propose that gastric acid suppression consequent to the use of PPI affects the immune system in a way that promotes infections [42]. One way the immune system is suppressed is by hampering cytotoxic T lymphocytes, natural killer cells, and polymorphonuclear neutrophil cell activities in the body [43]. PPI can also interfere with the signaling of tumor necrosis factor alpha (TNFα), IL-6, and nuclear factor kappa B (NFκB), and interleukin-1 beta (IL-1β) [44]. PPI also diminish bactericidal activity due to the neutrophil and monocyte inhibition they cause, coupled with intra and extra cellular reduction of nitric oxide and neutrophil reactive oxygen [45]. A cohort study conducted by Sheng Hong Lin et al. in 2021 has provided evidence that PPI have an influence on the gut microbiota [46]. The gut microbiota specifically can play a vital role in other enteric infections by boosting or restricting pathogen colonization [47]. Alteration of the microbiome, or host–microbiota interaction, has a major influence in how the host’s immune system develops and responds. Therefore, manipulation of the intestinal microbiota (which stems from the use of PPI) is considered an approach to inhibit the immune response of the body [48].

Certain limitations must be kept in mind when interpreting the findings of this study. A high heterogeneity was seen in the outcomes—this was expected due to methodological differences between studies, and a random-effects model was used in order to account for it. The methodological differences responsible for heterogeneity may include multiple features of the population such as age and gender, intervention factors such as PPI dosage, timing, or duration of usage before testing for COVID-19, the severity of COVID-19 infection, and wide variations in comorbidities (i.e., hypertension, diabetes, cardiovascular disease, renal disease, and respiratory disease). In addition, the COVID-19 protocols differed between countries which may have contributed to differences between studies that could not be accounted for in our results.

Moreover, reduced reliability of the included studies’ outcomes due to the variability in definitions of the severity of illness from COVID-19 also accounts for a higher heterogeneity. Due to the large sample size included by Israelsen et al. [15] and vast age group of participants catered by Seung Won Lee et al. [23], a higher heterogeneity in our secondary outcomes, that is, the incidence rate of COVID-19 infection and severity (ICU admission and death comparison) of COVID-19 infection in association with PPI usage, was encountered. Furthermore, as no clinical trials have been published, our meta-analysis only included observational studies and that constituted two problems that have led to the increase in heterogeneity: (1) studies did not have comparable follow-up times, (2) there were not enough studies to perform meta-regression. However, to avoid systematic errors and find the source of heterogeneity, we conducted a sensitivity analysis (i.e., removal of individual studies and observing any change in overall heterogeneity). It was observed that the removal of McKeigue et al. [27] led to a decrease in heterogeneity from 85% to 58%. This study was found to have a high bias due to various confounding variables such as age and the presence of comorbidities in the fatality group. Removal of this study changed the OR from 1.12 (95% CI 1.00, 1.25) to 0.92 (95% CI 0.80, 1.05). Thus, the finding of this study wherein PPI use was associated with an increased risk of mortality should be viewed as exploratory, and further studies are required to test this association.

## 5. Conclusions

PPI use was marginally associated with a nominal but statistically significant in-crease in the risk of COVID-19 infection. PPI use also increased the risk of the composite poor outcomes in patients with COVID-19.

In the ongoing COVID-19 pandemic, with newer variants emerging, it is crucial to know the factors influencing the risk of COVID-19. The increased risk of COVID-19 infection in PPI users appears to be only marginal and thus does not merit prophylactic discontinuation of PPI in patients for whom this medication is indicated. This study suggests that PPI increase the risk of poor clinical outcomes in patients with COVID-19; thus, PPI should be initiated with caution in this population. Moreover, patients with COVID-19 who are PPI users, should be monitored very closely for severe diseases. The current evidence is not sufficient to recommend discontinuation of PPI in patients with COVID-19. Further studies are required to consolidate out findings. Moreover, future studies should investigate whether the associations between PPI use and COVID-19 susceptibility and prognosis are influenced by the variant of COVID-19. The role of PPI and the duration of use should also be considered as important measures to take into account in these studies.

## Figures and Tables

**Table 1:** Characteristics of included studies.

Author, Year [Reference] from, Study Location	Study Design and Setting	No. of COVID-19 Tested Positive Patients	Exposure	Primary Outcome(s)
Elmunzer BJ et al., 2021 [22]; North America	Cohort	1846	PPI use within 1 month (after hospital admission)	Lack of association between PPI use and severity; No association between use of PPI and mortality
Almario CV et al., 2020 [16]; USA	Cohort	3386	PPI use once daily, and PPI use twice daily	Lack of association between PPI use and severity; No association between use of PPI and mortality
Lee SW et al., 2020 [23]; Korea	Cohort	4785	Current PPI use (within 90 days before the first positive SARS-CoV-2 RNA test and before hospitalization)	Severity (higher dose-PPI use and posthospitalization PPI use was significantly associated with severe COVID-19 symptoms)
Israelsen SB et al., 2021 [15]; Denmark	CaseControl	4473	Current PPI use (within 90 days before the first positive SARS-CoV-2 RNA test and before hospitalization)	Severity (hospital admission but not severe outcomes); No association found between PPI use and mortality
Liu JJ et al., 2021 [24]; MississiPPI	Cohort	56	PPI use once daily; PPI use twice daily	Mortality
Fan X et al., 2021 [19]; United Kingdom	Cohort	9469	PPI use (unspecified)	No significant association found between PPI use and mortality
Lee SW et al., 2020 [25]; Korea	Cohort	4785	Current PPI use and past PPI use	Composite endpoint 1 (requirement of oxygen therapy, intensive care unit admission, administration of invasive ventilation, or death); Composite endpoint 2 (severed clinical outcomes of COVID-19, intensive care unit admission, administration of invasive ventilation, or death)
Zhou J et al., 2021 [26]; China	Cohort	4445	Current PPI use and past PPI use	Severity
Ramchandran et al., 2020 [18]; USA	Cohort	295	PPI use (unspecified)	Severity (risk for developing acute respiratory distress syndrome); Mortality
McKeigue PM et al., 2020 [27]; Scotland	Matched Case Control	4251	Average daily doses of proton pump inhibitors	Severity and mortality (dose-response relationship was strongest in those PPI users aged less than 75 years)
Blanc F et al., 2020 [28], France	Retrospective CaseControl	89	Current PPI use and past PPI use	COVID-19 incidence (PPI use lowered the risk of COVID-19 infection)
Huh K et al., 2020 [29]; Korea	Case Control	7341	Past PPI use	No significant association found between PPI use and COVID-19 infection
Xiang Y et al., 2021 [17], China	Cohort	397,000	Current PPI use and past PPI use	Risk of COVID-19 Infection, severity, and mortality; Protective association between PPI use and COVID-19
Vila-Corcoles A et al., 2021 [30], Spain	Cohort	34,936	Current PPI use	No significant association found between PPI use and COVID-19 infection

**Table 2:** Baseline patient characteristics.

Study	Total Patients	Age	Males	Hypertension	Diabetes	Cardiovascular Disease	Renal Disease	Respiratory Disease
Elmunzer BJ et al., 2021 [22]; North America	1846	Mean 59.9 SD 16.4	1044 (56.6%)	1146 (62.1%)	658 (35.6%)	478 (25.9%)	175 (9.48%)	368 (20.0%)
Almario CV et al., 2020 [16]; America	3386	N/A	1168 (34.5%)	N/A	243 (7.2%)	N/A	N/A	N/A
Lee SW et al., 2020 [23]; Korea	4785	Mean 45.4 SD 18.8	1893 (44.7%)	945 (19.8%)	524 (11.0%)	263 (5.5%)	150 (3.1%)	523 (11.0%)
Israelsen SB et al., 2021 [15]; Denmark	4473	Median 60 IQR 48–73	1989 (44.5%)	N/A	564 (12.6%)	832 (18.6%)	231 (5.2%)	560 (12.6%)
Liu JJ et al., 2021 [24]; Mississippi	56	Mean 58 SD 14	20 (35.7%)	39 (69.6%)	24 (43.6%)	3 (5.4%)	7 (17.9%)	N/A
Fan X et al., 2021 [19]; United Kingdom	9469	>65	4611 (48.7%)	N/A	1226 (12.9%)	1741 (18.3%)	753 (7.9%)	1738 (18.3%)
Zhou J et al., 2021 [26]; China	4445	Median 44.8	307(58.6%)	209 (39.9%)	136 (26.0%)	136 (26.0%)	102 (19.5%)	516 (98.5%)
Lee SW et al., 2020 [25]; Korea	4785	≥18	N/A	N/A	N/A	N/A	N/A	N/A
Ramchandaran P et al., 2020 [18]; USA	295	>60	162 (54.9%)	209 (70.8%)	132 (44.7%)	45 (15.2%)	N/A	44 (14.9%)
McKeigue PM et al., 2021 [27]; Scotland	4251	N/A	N/A	N/A	949 (22.3%)	2649 (62.3%)	96 (2.3%)	1430 (33.6%)
Blanc F et al., 2020 [28]; France	89	Mean 84.4 SD 7.9	31 (34.8%)	61 (68.5%)	36 (40.4%)	53 (59.6%)	47 (52.8%)	15 (16.9%)
Huh K et al., 2020 [29]; South Korea	6507	N/A	2815 (43.3%)	1780 (27.4%)	1562 (24.0%)	1095 (16.8%)	749 (11.5%)	2893 (44.5%)
Xiang Y et al., 2021 [17]; United Kingdom	397,000	Mean 68.1 SD 8.1	177,441 (44.7%)	131,180 (33.0%)	28,287 (7.1%)	31,258 (7.9%)	N/A	69,397 (17.4%)
Vila-Corcoles A et al., 2021 [30]; Spain	205	N/A	83 (40.5%)	N/A	69 (33.7%)	85 (41.5%)	39 (19.0%)	42 (20.5%)

**Table 3:** Patient proton pump inhibitor (PPI) use.

	Current		Prior
Elmunzer BJ et al., 2021 [22]; North America	Once daily	N/A	N/A
	Twice daily	N/A	N/A
	N/A	417 (Within one month of hospital admission)	N/A
Almario CV et al., 2020 [16]; America	Once daily	2436	N/A
	Twice daily	198	N/A
	N/A	N/A	N/A
Lee SW et al., 2021 [23]; Korea	Once daily	N/A	N/A
	Twice daily	N/A	N/A
	N/A	364 (Within 30 days before first COVID test)	188 (More than 30 days, up to a year before first COVID test)
Israelsen SB et al., 2021 [15]; Denmark	Once daily	N/A	N/A
	Twice daily	N/A	N/A
	N/A	4473 (Within 90 days before positive COVID test, before hospitalization)	19338 (More than 90 days before positive COVID test, before hospitalization)
Liu JJ et al., 2021 [24]; Mississippi	Once daily	26	N/A
	Twice daily	5	N/A
	N/A	N/A	N/A
Fan X et al., 2021 [19]; United Kingdom	Once daily	N/A	N/A
	Twice daily	N/A	N/A
	N/A	250	N/A
Zhou J et al., 2021 [26]; China	Once daily	N/A	N/A
	Twice daily	N/A	N/A
	N/A	524	N/A
Lee SW et al., 2021 [25]; Korea	Once daily	489	N/A
	Twice daily (or more)	312	N/A
	N/A	801	N/A
Ramchandaran P et al., 2020 [18]; USA	Once daily	N/A	N/A
	Twice daily	N/A	N/A
	N/A	46	N/A
McKeigue PM et al., 2020 [27]; Scotland	Once daily	1743	N/A
	Twice daily	239	N/A
	N/A	N/A	N/A
Blanc F et al., 2020 [28]; France	Once daily	N/A	N/A
	Twice daily	N/A	N/A
	N/A	23	N/A
Huh K et al., 2020 [29]; South Korea	Once daily	N/A	N/A
	Twice daily	N/A	N/A
	N/A	851	N/A
Xiang Y et al., 2021 [17]; United Kingdom	Once daily	N/A	N/A
	Twice daily	N/A	N/A
	N/A	8086	N/A
Vila-Corcoles A et al., 2020 [30]; Spain	Once daily	N/A	N/A
	Twice daily	N/A	N/A
	N/A	99	N/A

## Supplementary Materials

The following supporting information can be downloaded at https://www.mdpi.com/article/10.3390/tropicalmed7030037/s1, Table S1: Search Strategy used in each database searched; Table S2: Quality assessment of included observational studies using Newcastle-Ottawa Scale.
