# Supplementary material for: The Use of Proton Pump Inhibitors and COVID-19: A Systematic Review and Meta-Analysis
Source: Trop Med Infect Dis. 2022 Feb 28;7(3):37. doi: 10.3390/tropicalmed7030037 (PMC8950138; doi:10.3390/tropicalmed7030037)
Supplement: Supplementary file 1 [file tropicalmed-07-00037-s001.zip › tropicalmed-1534261-supplementary.pdf]

**SUPPLEMENTARY MATERIAL**

**Table S1**. Search strategy used in each database searched

| Database (Articles Retrieved) | Search Strategy |
|---|---|
| Pubmed (98 articles) | ("sars cov 2"[MeSH Terms] OR "sars cov 2"[All Fields] OR "sars cov 2"[All Fields] OR ("sars cov 2"[MeSH Terms] OR "sars cov 2"[All Fields] OR "covid"[All Fields] OR "covid 19"[MeSH Terms] OR "covid 19"[All Fields]) OR ("coronavirus"[MeSH Terms] OR "coronavirus"[All Fields] OR "coronaviruses"[All Fields]) OR ("covid 19"[All Fields] OR "covid 19"[MeSH Terms] OR "covid 19 vaccines"[All Fields] OR "covid 19 vaccines"[MeSH Terms] OR "covid 19 serotherapy"[All Fields] OR "covid 19 serotherapy"[Supplementary Concept] OR "covid 19 nucleic acid testing"[All Fields] OR "covid 19 nucleic acid testing"[MeSH Terms] OR "covid 19 serological testing"[All Fields] OR "covid 19 serological testing"[MeSH Terms] OR "covid 19 testing"[All Fields] OR "covid 19 testing"[MeSH Terms] OR "sars cov 2"[All Fields] OR "sars cov 2"[MeSH Terms] OR "severe acute respiratory syndrome coronavirus 2"[ALl Fields] OR "ncov"[All Fields] OR "2019 ncov"[All Fields] OR (("coronavirus"[MeSH Terms] OR "coronavirus"[All Fields] OR "cov"[All Fields]) AND 2019/11/01:3000/12/31[Date - Publication])) OR ("sars cov 2"[MeSH Terms] OR "sars cov 2"[All Fields] OR "ncov"[All Fields]) OR "Novel Coronavirus"[All Fields]) AND (("Proton Pump Inhibitors"[All Fields] OR "PPI"[All Fields] OR (("reflux"[All Fields] OR "refluxant"[All Fields] OR "refluxate"[All Fields] OR "refluxates"[All Fields] OR "refluxed"[All Fields] OR "refluxers"[All Fields] OR "refluxes"[All Fields] OR "refluxing"[All Fields] OR "refluxive"[All Fields]) AND ("medic"[All Fields] OR "medical"[All Fields] OR "medicalization"[MeSH Terms] OR "medicalization"[All Fields] OR "medicalizations"[All Fields] OR "medicalize"[All Fields] OR "medicalized"[All Fields] OR "medicalizes"[All Fields] OR "medicalizing"[All Fields] OR "medically"[All Fields] OR "medicals"[All Fields] OR "medicated"[All Fields] OR "medication s"[All Fields] OR "medics"[All Fields] OR "pharmaceutical preparations"[MeSH Terms] OR ("pharmaceutical"[All Fields] AND "preparations"[All Fields]) OR "pharmaceutical preparations"[All Fields] OR "medication"[All Fields] OR "medications"[All Fields]))) AND ("mortality"[MeSH Terms] OR "mortality"[All Fields] OR "mortalities"[All Fields] OR "mortality"[MeSH Subheading] OR ("hospital mortality"[MeSH Terms] OR ("hospital"[All Fields] AND "mortality"[All Fields]) OR "hospital mortality"[All Fields]) OR "sever*"[All Fields] OR "Patient Acuity"[All Fields] OR "Severity of Illness Index"[All Fields] OR ("Intensive Care Units"[MeSH Terms] OR ("intensive"[All Fields] AND "Care"[All Fields] AND "units"[All Fields]) OR "Intensive Care Units"[All Fields] OR "icu"[All Fields]) OR "Intensive Care Units"[All Fields] OR ("ventilators, mechanical"[MeSH Terms] OR ("ventilators"[All Fields] AND "mechanical"[All Fields]) OR "mechanical ventilators"[All Fields] OR ("mechanical"[All Fields] AND "ventilator"[All Fields]) OR "mechanical ventilator"[All Fields]) OR (("medicine"[MeSH Terms] OR "medicine"[All Fields] OR "specialty"[All Fields] OR "special"[All Fields] OR "specialisations"[All Fields] OR "specialise"[All Fields] OR "specialised"[All Fields] OR "specialises"[All Fields] OR "specialising"[All Fields] OR "specialisms"[All Fields] OR "specialities"[All Fields] OR "speciality"[All Fields] OR "specialization"[MeSH Terms] OR "specialization"[All Fields] |

| | |
|---|---|
| OR "special | isation"[All Fields] OR "specialism"[All Fields] OR "specializations"[All Fields] OR "specialize"[All Fields] OR "specialized"[All Fields] OR "specializes"[All Fields] OR |

| | |
|---|---|
| | "specializing"[All Fields] OR "specially"[All Fields] OR "specials"[All Fields] OR "specialties"[All Fields] OR "specialty s"[All Fields]) AND "Care"[All Fields]) OR ("epidemiology"[MeSH Subheading] OR "epidemiology"[All Fields] OR "morbidity"[All Fields] OR "morbidity"[MeSH Terms] OR "morbid"[All Fields] OR "morbidities"[All Fields] OR "morbids"[All Fields] |
| CINAHL (33 articles) | ((SARS-CoV-2) OR (COVID) OR (Coronavirus) OR (COVID-19) or (nCov) OR (“Novel Coronavirus”)) AND ((“Proton Pump Inhibitors”) OR (PPI) OR (Reflux Medications) AND ((Mortality) OR (Hospital Mortality) OR (Sever*) OR (“Patient Acuity”) OR (“Severity of Illness Index”) OR (ICU) OR (“Intensive Care Units”) OR (Mechanical Ventilator) OR (Special Care) OR (Morbidity)) |
| Science Direct (156 articles) | SARS-CoV-2 or COVID-19 or nCov or AND “Proton Pump Inhibitors” or PPI AND Mortality OR Hospital Mortality OR Severity |

**Table S2. Quality assessment of included observational studies using Newcastle-Ottawa Scale**

| Study/ Score | Selection | | | | Comparability | | Outcome / Exposure | | | Total Score (9) | Quality (High, Moderate, Low) |
|---|---|---|---|---|---|---|---|---|---|---|---|
| | S1 | S2 | S3 | S4 | C1 | | O1 | O2 | O3 | | |
| Elmunzer BJ et al. | * | * | * | | * | * | * | * | * | 8 | High |
| Almario CV et al. | * | * | * | | * | | * | * | * | 7 | Moderate |
| Lee SW et al. | * | * | * | | * | | * | * | * | 7 | Moderate |
| Israelsen SB et al. | * | * | * | | * | | * | * | * | 7 | Moderate |
| Liu JJ et al. | * | * | * | | * | * | * | * | * | 8 | High |
| Fan X et al. | * | * | * | * | * | * | * | * | * | 9 | High |
| Lee SW et al. | * | * | * | * | * | * | * | * | * | 9 | High |
| Zhou J et al. | * | * | * | * | * | | * | * | | 7 | Moderate |
| Ramchandran P et al. | * | * | * | * | * | | * | * | | 7 | Moderate |
| McKeigue PM et al. | * | * | * | * | * | * | * | * | * | 9 | High |
| Blanc E et al. | * | * | * | * | * | | * | * | * | 8 | High |
| Huh K et al. | * | * | * | * | | | * | * | | 6 | Moderate |
| Xiang Y et al. | * | * | * | * | * | * | * | * | * | 9 | High |
| Vila-Corcoles A et al. | * | * | * | * | * | * | * | * | * | 9 | High |
